# One-Step Synthesis of SnO_2_/Carbon Nanotube Nanonests Composites by Direct Current Arc-Discharge Plasma and Its Application in Lithium-Ion Batteries

**DOI:** 10.3390/nano11113138

**Published:** 2021-11-21

**Authors:** Da Zhang, Yuanzheng Tang, Chuanqi Zhang, Qianpeng Dong, Wenming Song, Yan He

**Affiliations:** College of Electromechanical Engineering, Qingdao University of Science and Technology, Qingdao 266061, China; qustzd17863928637@163.com (D.Z.); tangyuanzheng@163.com (Y.T.); qustzcq@163.com (C.Z.); 13593574106@163.com (Q.D.); swmpower@163.com (W.S.)

**Keywords:** tin oxide, carbon nanotube, direct current arc-discharge plasma, lithium-ion batteries, anode materials

## Abstract

Tin dioxide (SnO_2_)-based materials, as anode materials for lithium-ion batteries (LIBs), have been attracting growing research attention due to the high theoretical specific capacity. However, the complex synthesis process of chemical methods and the pollution of chemical reagents limit its commercialization. The new material synthesis method is of great significance for expanding the application of SnO_2_-based materials. In this study, the SnO_2_/carbon nanotube nanonests (SnO_2_/CNT NNs) composites are synthesized in one step by direct current (DC) arc-discharge plasma; compared with conventional methods, the plasma synthesis achieves a uniform load of SnO_2_ nanoparticles on the surfaces of CNTs while constructing the CNTs conductive network. The SnO_2_/CNT NNs composites are applied in LIBs, it can be found that the nanonest-like CNT conductive structure provides adequate room for the volume expansion and also helps to transfer the electrons. Electrochemical measurements suggests that the SnO_2_/CNT NNscomposites achieve high capacity, and still have high electrochemical stability and coulombic efficiency under high current density, which proves the reliability of the synthesis method. This method is expected to be industrialized and also provides new ideas for the preparation of other nanocomposites.

## 1. Introduction

Lithium-ion batteries (LIBs), as the growing popular power sources, have attracted considerable attention in portable electronic devices and are attractive to power electric vehicles [1,2,3,4,5].Therefore, developing new electrode materials and optimizing the preparation process are still hot issues in current research. To circumvent the low theoretical capacity (~372 mAh g^−1^), low energy and power density of traditional commercial graphite [6] and meet the demands of better performance (higher power and energy density, super-long cycle life and more excellent cycle stability) of LIBs, extensive efforts have been devoted to find new anode materials which have higher theoretical capacity and better cycling performance [7,8], such as silicon [9], metal oxides [10,11,12], alloys [13,14], carbon-based composite materials [15,16,17,18], etc.

Among the above alternative anode materials, in the past few years, SnO_2_-based materials have attracted growing research attention due to their suitable charge/discharge voltage range, high theoretical specific capacity (~782 mAh g^−1^), low toxicity and cost [19], meanwhile, various structures and preparation processes have been explored to remove the biggest bottlenecks that great volume change (>200%) upon the large amount of lithium insertion/extraction into/from SnO_2_ and prevent the pulverization of SnO_2_ [20,21]. Studies have shown that an effective way to adapt to volume changes and maintain the mechanical integrity of composite electrodes is to uniformly disperse SnO_2_ nanoparticles in a conductive matrix (especially carbon materials) [19,20,21]. Liu et al. [19] synthesized a new type of SnO_2_ nanorod structure grown on graphite by hydrothermal method; the results showed that the SnO_2_/graphite composite maintained higher capacity and better cycling stability than graphite. Wang et al. [20] prepared carbon-coated SnO_2_/C nanocomposites by a two-step hydrothermal route, which exhibited a markedly improved cycling performance. Chen et al. [22] fabricated the SnO_2_-reduced graphene oxide-carbon nanotube composites by a facile one-step microwave-assisted method, the electrochemical tests showed that the SnO_2_-RGO-CNT composite with 60 wt.% SnO_2_ maintained a maximum capacity of 502 mAh g^−1^ after 50 cycles at 100 mA g^−1^. Although the above studies have effectively alleviated the volume change upon the large amount of lithium insertion/extraction into/from SnO_2_, SnO_2_-based materials have not achieved sustained development in LIBs, the complexity of the chemical preparation process and the contamination of chemical reagents are also important reasons that limit their commercialization besides the discovery of new high-performance electrode materials. Moreover, society’s initiatives for green and environmental protection and the urgent desire to accelerate the process of industrialization have driven the preparation of materials to a green and rapid transition. Therefore, developing new material synthesis methods is of great significance to expand the application of SnO_2_/CNT composites.

In recent years, plasma technology has attracted widespread attention in the preparation and treatment of nanocomposites due to its simple operation, fast synthesis and environmental friendliness [23,24]. Various metal oxides including nano-SnO_2_ have been successfully prepared by the plasma method [25,26,27], which greatly avoided these problems including the complexity of chemical methods and the use of chemical reagents. However, the compounding process of SnO_2_ nanoparticles and conductive matrix is often another process, which poses a higher challenge in the structural design and synthesis process of SnO_2_-based composite materials.In previous works, the DC arc-discharge plasma has shown excellent effects in realizing the dispersion of CNTs [28,29], providing a basis for the design of CNTs conductive matrix structure, and the CNTs conductive network constructed by plasma showed excellent conductivity in conductive films in our research [30], it is bound to promote its application in LIBs. Besides, instantly loading SnO_2_ nanoparticles over the surfaces of CNTs followed by the construction of CNTs conductive network structure can not only buffer the volume expansion of SnO_2_ nanoparticles and realize the synergy of two materials, but promote its industrialization due to the simple and green preparation process.

Herein, the SnO_2_/CNT nanonests (NNs) composites are first synthesized in one step by DC arc-discharge plasma, in this process, the dispersion of CNTs and the loading of SnO_2_ nanoparticles are realized simultaneously, which overcomes the complex preparation of traditional chemical methods and the pollution of chemical reagents. Moreover, the nanonest-like conductive structure provides large space for volume change, and also enhances electron transfer between the electrode and SnO_2_ during lithium ions insertion/extraction process. This method is expected to be industrialized and also provides new ideas for the preparation of other nanocomposites. 

## 2. Materials and Methods

### 2.1. Synthesis of SnO_2_/CNTs NNs Composites

**Figure 1:**
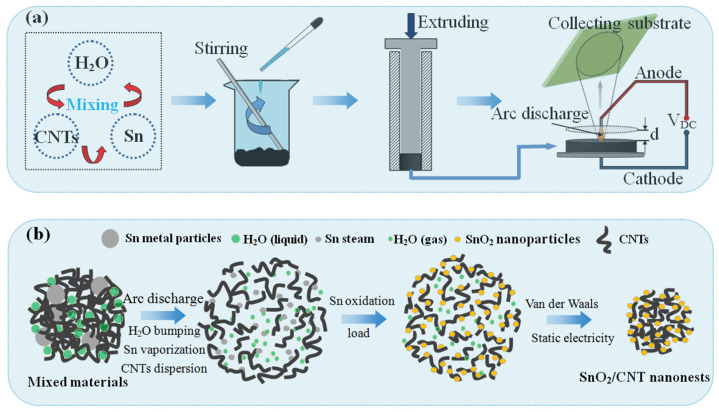
Schematic diagram of the preparation process of SnO_2_/CNT NNs composites. (**a**) macro-preparation process; (**b**) micro-composite mechanism.

The schematic diagram of the synthetic route of SnO_2_/CNT NNs composites is shown in Figure 1a. First of all, micron-sized Sn particles (99.999% purity), CNTs (GT-400; length: 3–12 μm, diameter: 20–30 nm; Shandong Dazhan Nano Materials co. ltd.) and deionized water (H_2_O) were initially mixed according to a mass ratio of 8:1:5, and stirred through a glass rod for 30 min until uniform, in this process, additional H_2_O can be added appropriately to further adjust the viscosity and uniformity of the mixture. Later, with a metal wolfram rod as anode electrode, the prepared mixtures were squeezed into a dense cylindrical electrode as cathode and moved onto a conductive substrate made of copper to keep a certain distance (d) between two electrodes, and d = 2 mm in this experiment. High-voltage direct current (V_h_ = 10,000 V) was applied between the two electrodes, and the DC arc-discharge plasma was generated while the air was broken down; the mixture as the cathode will produce strong local dispersion under the action of the DC arc-discharge plasma. The hot gas steam pushed the dispersed mixtures upward to the collecting substrate, and finally adhered on the collecting substrate through the electrostatic interaction and significant van der Waals force shown by the nanomaterials [29].

In the micro-composite mechanism of SnO_2_/CNTs NNs as shown in Figure 1b, under the DC arc-discharge plasma, the H_2_O in the mixture was instantly bumping and the volume expansion force was generated, thereby forming the large pressure gradient between the agglomerated CNTs, which forced the CNTs to be dispersed. Simultaneously, due to the low melting point (232 °C), the micron-sized Sn particles were vaporizing under the action of plasma thermal excitation, and forming the Sn steam zone near the dispersed CNTs. Furthermore, Sn was oxidized to SnO_2_ thanks to the oxidizing active substance in the plasma, and uniformly loaded over the surfaces of CNTs, forming the dispersed SnO_2_/CNT composites structure. Finally, with the diffusion of SnO_2_/CNT and collection on the collecting substrate, secondary agglomeration occurred due to van der Waals force and static electricity [31], and the SnO_2_/CNT NNs composites were obtained.

### 2.2. Material Characterizations

The morphology was characterized by using a field-emission scanning electron microscopy (SEM) (Hitachi, Tokyo, Japan, SU8010). A transmission electron microscopy (TEM) (Hitachi, Tokyo, Japan, H-8100) was adopted to characterize the further detailed microstructure. Crystallite size determination and phase identification were carried out on an X-ray Diffractometer (XRD) (Rigaku, Tokyo, Japan, Ultima IV) with Cu/Ka radiation (k = 1.5406 Å). The Raman spectroscopy (Renishaw, Shanghai, China) with a 532 nm laser line was applied to characterize the crystallinities of the pristine CNTs and SnO_2_/CNT NNs composites obtained by DC arc-discharge plasma. The chemical compositions were further characterized by adopting an X-ray photoelectron spectroscopy (XPS) analysis under ultra-high vacuum using a Thermo ESCALAB 250Xi device employing an Al-Ka (hv = 1253.6 eV) excitation source. Thermogravimetric analysis (TGA) was carried out by using a thermogravimetric analyzer (Netzsch, Selb, Germany, TG 209 F1) with a heating rate of 15 °C min^−1^ in air. In addition, The N_2_ adsorption/desorptiontest was estimated by using specific surface and pore size analysis instrument (BET, BSD-PS1, Beijing, China).

### 2.3. Electrochemical Measurements

For electrochemical measurement, SnO_2_/CNT NNs, conductive carbon black, and polyvinylidene fluoride (PVDF), with a weight ratio of 8:1:1, were dissolved in N-methyl pyrrolidinone (NMP) and mixed together thoroughly to form slurry. Then, the resultant slurries were coated onto copper foil substrates. Finally, the working electrodes were dried at 120 °C under vacuum for 12 h. Polypropylene film and Li metals were used as separator and counter anode, respectively, and the 1.15 M LiPF_6_ electrolyte solution dissolved in a mixture of ethylene carbonate/diethyl carbonate (1:1, vol.%) was electrolyte. The electrochemical measurements were tested using a Battery Testing System (Ningbo baite testing equipment Co., Zhejiang, China). Cyclic voltammetry (CV) curves were collected on a CHI660D electrochemical workstation at 0.2 mV s^−1^ within the voltage range of 0.01–3.00 V and electrochemical impedance spectroscopy (EIS) was performed at 23 °C from 0.01 Hz to 100 KHz with a perturbation amplitude of 5 mV. 

## 3. Results and Discussion

### 3.1. Microstructure and Morphology of SnO_2_/CNT NNs Composites

**Figure 2:**
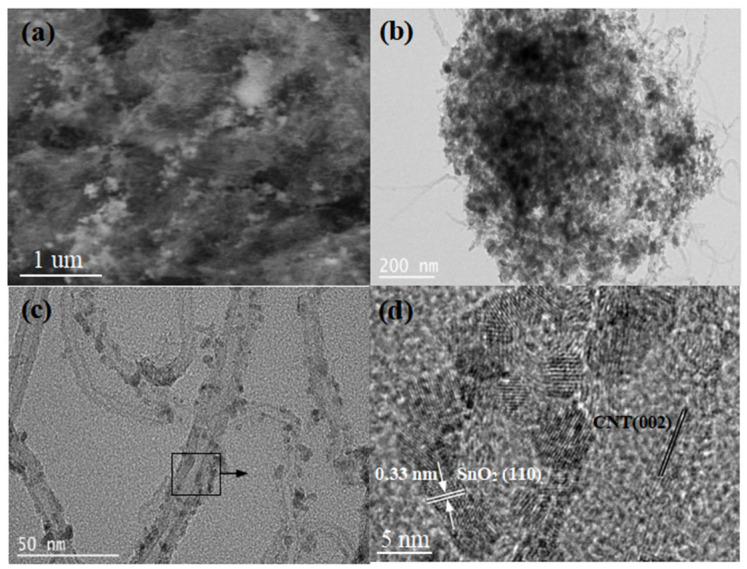
(**a**) SEM, (**b**,**c**) TEM and (**d**) HRTEM images of SnO_2_/CNT NNs composites.

The microstructure and morphology of SnO_2_/CNT NNs composites are shown in Figure 2. Figure 2a shows a typical SEM image of the SnO_2_/CNT NNs composites, it can be clearly seen that SnO_2_ nanoparticles are uniformly embedded in dispersed CNTs conductive network, which is attributed to the vaporization and oxidation process of metallic Sn and the construction of the dispersed CNTs conductive network under the action of DC arc-discharge plasma. More clearly, Figure 2b,c depicts the TEM images of SnO_2_/CNT NNs composites, in which the SnO_2_ nanoparticles are densely anchored on the surfaces of CNTs and the average particle size is approximately 5 nm. The overlapping CNTs form a dense nanonest-like conductive network structure, which is conducive to the transmission of electrons, besides, the unique nanonest-like conductive network structure will provide a large void space and mechanical support to relieve the volume change and strain caused upon the alloying/dealloying of SnO_2_, thereby preventing the pulverization of SnO_2_ nanoparticles. The HRTEM image in Figure 2d shows lattice fringes with a pitch of 0.33 nm, which corresponds to the interplanar distance of the (1 1 0) planes in rutile SnO_2_ [32], meanwhile, it can be clearly seen that the lattice fringes of CNTs correspond to the interplanar distance of the (0 0 2) planes. 

**Figure 3:**
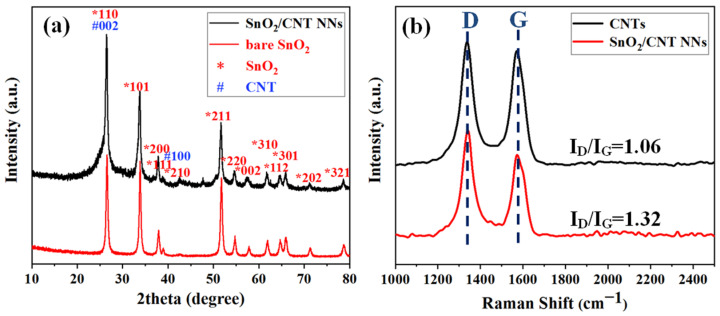
(**a**) XRD patterns of bare SnO_2_, SnO_2_/CNT NNs composites and (**b**) Raman spectra of pristine CNTs, SnO_2_/CNT NNs composites.

The XRD patterns of bare SnO_2_ and SnO_2_/CNT NNs composites are shown in Figure 3a. The red line shows the main diffraction peaks of SnO_2_, by comparison with the standard values (JCPS No. 21-1272), it is confirmed that the principal diffraction peak has a good correspondence with the tetragonal rutile phase of SnO_2_. The black line shows that the peak positions assigned to SnO_2_ indexed well with the positions of the bare SnO_2_. Besides, the (1 1 0) and (2 1 0) reflection of SnO_2_ is overlapped by the (0 0 2) and (1 0 0) reflection of CNTs, respectively. 

In order to explore the influence of DC arc-discharge plasma on the structure of CNTs, the structures of the CNTs were analyzed by Raman spectra, as shown in Figure 3b. The Raman spectrum of pristine CNTs were composed of two strong peaks at 1335 cm^−1^ and 1572 cm^−1^, corresponding to the D and G bands, respectively. The D band constitutes a disordered induction characteristic, which is derived from the vibration of C atoms with dangling bonds, while the G band is derived from the tangential shear mode in C atoms, which corresponds to the tensile mode in the graphite plane [33,34]. The lower intensity of D/G band intensity ratio (I_D_/I_G_) reflects the higher degree of graphitization; the ratio of the intensities (I_D_/I_G_) was 1.06 for the pristine CNTs and 1.32 for the SnO_2_/CNT NNs, which suggested that there was a certain degree of damage to the CNTs structure in the process of preparing SnO_2_/CNT NNs by the DC arc-discharge plasma, which is consistent with our previous researches [31]. The defects on the CNTs walls may cause many cavities and alleyways in the graphite layers, which is beneficial to enhancing the anchoring effect of SnO_2_ nanoparticles and CNTs, and also provides more reaction sites for Li^+^. Besides, CNTs still have a high degree of graphitization and thus retain high electrical conductivity.

**Figure 4:**
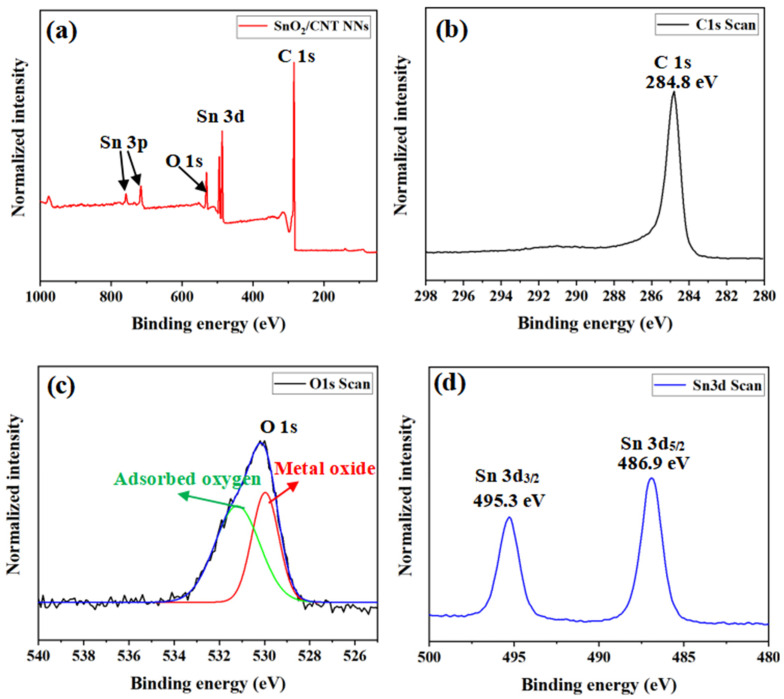
(**a**) XPS spectral survey of the SnO_2_/CNT NNs composites. (**b**) C 1s, (**c**) O 1s and (**d**) Sn 3d spectrum obtained from the SnO_2_/CNT NNs composites.

The XPS studies were carried out to identify the chemical composition of SnO_2_/CNT NNs composites. Figure 4a shows the XPS survey spectra of SnO_2_/CNT NNs composites, it can be clearly seen that the SnO_2_/CNT NNs composites produced peaks corresponding to O 1s, C 1s, Sn 3d as well as Sn 3p, indicating the presence of Sn in the sample besides CNTs and SnO_2_, this is due to the fact that a small amount of Sn was not oxidized during the preparing process by plasmas. Figure 4b–d illustrate the spectra for C, O and Sn elements, respectively. The binding energy of 284.8 eV for C 1s mainly corresponds to the carbon atoms in CNTs (Figure 4b). The peaks in Figure 4c correspond to the O spectrum with different chemical states. The peak close to 530 eV could be assigned to O in SnO_2_, while peak at around 532.3 eV can be assigned to O in H_2_O or adsorbed oxygen. In Figure 4d, the Sn 3d spectrum obtained from the SnO_2_/CNT NNs composites exhibited binding energies of 495.3 eV for Sn 3d_3/2_ and 486.9 eV for Sn 3d_5/2_, which confirmed that the rutile SnO_2_ nanoparticles were anchored on the surface of CNTs.

**Figure 5:**
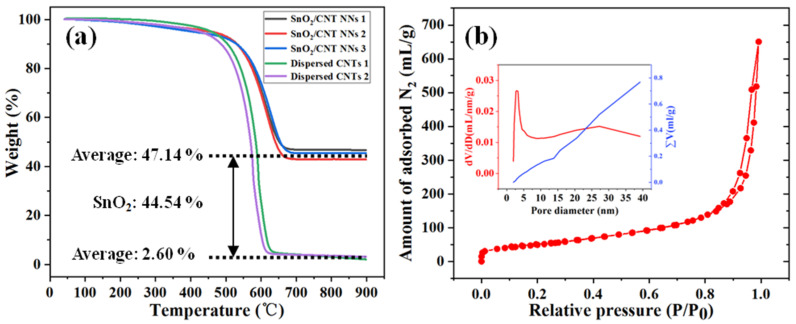
(**a**) TGA curve of SnO_2_/CNT NNs composites in air. flow rate 20 mL min^−1^, heating rate 15 °C min^−1^, (**b**) N_2_ adsorption/desorption isotherm of the SnO_2_/CNT NNs composites, inset shows the porosity distribution by the Barrett-Joyner-Halenda (BJH) method.

TGA analysis was used to identify the composition and thermal/chemical stability of the SnO_2_/CNT NNs composites, average of multiple measurements were adopted to ensure the accuracy of SnO_2_ content, meanwhile, considering the refractory impurities contained in CNTs, the CNTs (without Sn) dispersed by the DC arc-discharge plasma were used as the benchmark to value the content of SnO2, as shown in Figure 5a. It can be seen that the residual content of the same sample under different tests is very stable, the average residual content of SnO_2_/CNT NNs composites and dispersed CNTs are 47.14% and 2.60%, respectively, thus the content of SnO_2_ in the sample can be calculated to be 44.54%. Moreover, the SnO_2_/CNT NNs composites have higher thermal stability than dispersed CNTs.

The porous structure of the SnO_2_/CNT NNs composites was characterized by N_2_ adsorption/desorption measurement. As shown in Figure 5b, the adsorption isotherm and pore size distribution analyzed by using the Barrett-Joyner-Halenda (BJH) method. The BET specific surface area of the SnO_2_/CNT NNs composites is 181.92 m^2^ g^−1^, and the pore volume is 0.89 mL g^−1^. The average pore diameter of BJH is 16.76 nm. The abundant pore structure and large specific surface are conducive to alleviate strain, enhance electron-electronic contact area and improve the kinetics.

### 3.2. Electrochemical Performance of SnO_2_/CNT NNs as Anode Materials in LIBs

**Figure 6:**
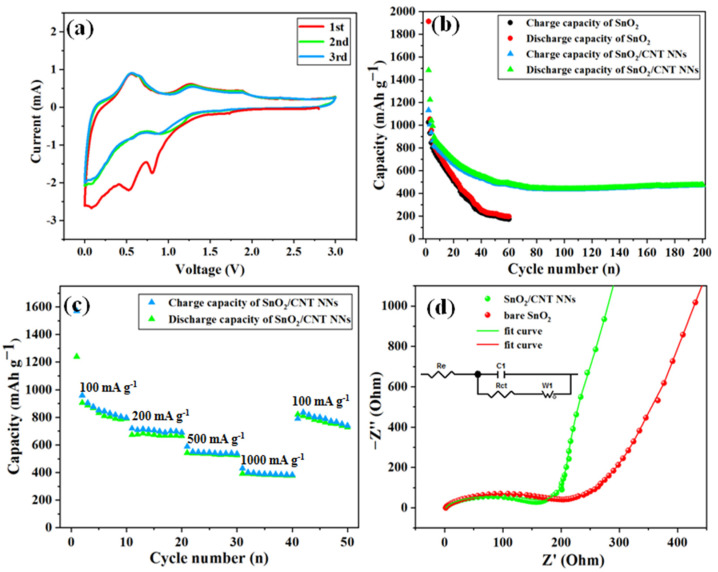
(**a**) CV curves of the SnO_2_/CNT NNs composites at a scanning rate of 0.2 mV s^−1^, (**b**) cycling performance at 0.01−3 V and 100 mA g^−1^ of the SnO_2_/CNT NNs composites and rare SnO_2_, (**c**) the rate performance of the SnO_2_/CNT NNs composites at various current densities, and (**d**) EIS spectra of the bare SnO_2_ and SnO_2_/CNT NNs composites at 25 ℃ from 0.1 to 100 kHz.

The electrochemical behavior of SnO_2_/CNT NNs composites was evaluated by CV as shown in Figure 6a. The CV curves of SnO_2_/CNT NNs composites in the first three cycles represents the reaction process of SnO_2_ and CNTs during the cycle. In the first cycle, the strong reduction peak appears at 0.8 V in the first cycle, which can be attributed to the reduction in SnO_2_ during the reaction and the formation of a solid electrolyte interphase (SEI) layer [35], and it also can be found with a lower intensity in the second cycle. The peak close to 0.01 V may be attributed to the formation of LiC_6_ induced by Li intercalation into CNTs, and other reduction peaks (0.01–0.8 V) can be attributed to the formation of Li_x_Sn [36]. In addition, the peaks at 0.2 V and 0.5 V can be ascribed to deintercalation of LiC_6_ and the dealloying of Li_x_Sn, respectively [35], and there is a weak oxidation peak at 1.23 V, which could be attributed to the partly reversible reaction from Sn to SnO_2_ [37] and the unoxidized Sn within the SnO_2_/CNT NNs composites confirmed by Figure 4.

Figure 6b compares the charge-discharge cycle performance of bare SnO_2_ and SnO_2_/CNT NNs composites, it can be seen that the bare SnO_2_ particles have an initial discharge and charge capacity of 1914.3 and 1026.7 mAh g^−1^, respectively. The initial coulomb efficiency is only 53 % comparable with that expected for SnO_2_ anodes, which is mainly attributed to the formation of Li_2_O and SEI layer. Although the bare SnO_2_ exhibits a high initial discharge capacity, the capacity rapidly declines to below 200 mAh g^−1^ after 60 cycles, displaying poor cycle stability of bare SnO_2_. By contrast, the SnO_2_/CNT NNs composites shows excellent cycle stability except for the obvious capacity decay in the initial cycle, achieving a capacity of 472 mAh g^−1^ after 200 cycles at 100 mA g^−1^. Besides, the initial coulomb efficiency of SnO_2_/CNT NNs composites can reach up to 76 %.

In order to further investigate the rate performances of SnO_2_/CNT NNs composites, the cycle rate gradually increasing from 100 mA g^−1^ to 1000 mA g^−1^ and then reversing to 100 mA g^−1^, was adopted as shown in Figure 6c. It can be seen that the SnO_2_/CNT NNs composites exhibit excellent cycling performance even at the high cycling rate of 1000 mA g^−1^, the reversible discharge capacity is still preserved at 395 mAh g^−1^ and the coulombic efficiency is around 98.6 % after 40 cycles at different current densities. Although the Coulomb efficiency is slightly reduced (96.5%), when reversing the cycling rate from 1000 mA g^−1^ to 100 mA g^−1^, the SnO_2_/CNT NNs composites show strong recovery ability of capacity. The good rate capability can be associated with the nanonest-like structure, the overlapping CNTs provide mechanical support to achieve good electrical contact between the CNTs and SnO_2_ nanoparticles and can be conducive to the transmission of electrons due to the high electronic contact area ensured by the large specific surface area.

Figure 6d displays the EIS spectra of the bare SnO_2_ and SnO_2_/CNT NNs composites, both are composed of a semicircle in high frequencies and a diagonal line in low frequencies. Based on the equivalent circuit, the EIS spectra are fitted as shown in the inset of Figure 6d. It can be seen that the fitting curves are well consistent with the EIS of both electrodes, respectively. In high frequencies, the kinetic resistance of charge transfer at the electrode–electrolyte interface is represented by Rct, the fitting results show that the R_ct_ of SnO_2_/CNT NNs composites is 119.8 Ω, which is lower than the 198.7 Ω of the bare SnO_2_, indicating that the introduction of CNTs accelerate electron transport during the electrochemical reaction and it has a higher charge transfer efficiency. Meanwhile, in low frequencies, the slope of the line represents the ionic conductivity of materials, the impedance slope of the SnO_2_/CNT NNs composites is greater than that of the bare SnO_2_, indicating that the SnO_2_/CNT NNs composite has an excellent Li+ diffusion rate. This can explain why SnO_2_/CNT NNs composites have better lithium storage characteristics than the bare SnO_2_ and exhibit better electrochemical performance.

**Figure 7:**
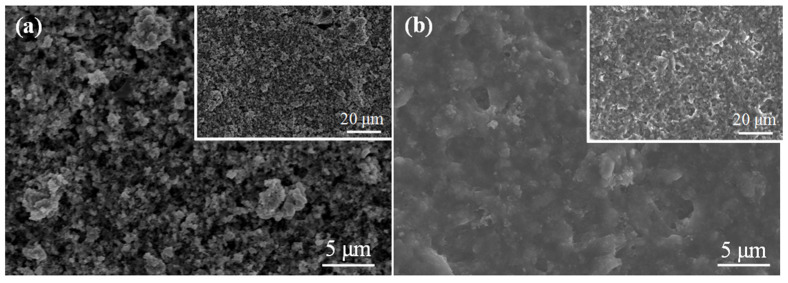
SEM after 200 cycles of (**a**) SnO_2_ and (**b**) SnO_2_/CNT NNs electrode.

Figure 7 exhibits the SEM images after 200 cycles of SnO_2_ and SnO_2_/CNT NNs electrode, it can be clearly seen that the surface of SnO_2_ electrode is rugged and shows serious volume change. In the contrary, the surface of SnO_2_/CNT NNs electrode is flat and smooth, and there is no obvious volume change, which is because the unique nanonest-like structure provides adequate room for the volume expansion, this is why the SnO_2_/CNT NNs electrode shows excellent electrochemical performance.

Based on the above results and discussion, the advantages of the plasma one-step synthesis technology and the stable cycle performance of SnO_2_/CNT NNs composites are attributed to the following points: (1) plasma one-step synthesis is to achieve a uniform load of SnO_2_ nanoparticles while constructing CNTs conductive network, which saves time and energy; (2) this synthesis does not involve any chemicals and it is more environmentally friendly compared with conventional methods; (3) the overlapping CNTs form a dense nanonest-like conductive network structure, which is conducive to the transmission of electrons and ensures the excellent electron contact between Li^+^ and SnO_2_; (4) the defects on the CNTs walls generated under the action of plasma may create many cavities and channels in graphite layers, providing more reaction sites for Li^+^; (5) nanonest-like pore structure provides adequate room for the volume expansion, allowing stable cycle performance by preventing SnO_2_ nanoparticles pulverization.

All in all, these results suggest that the SnO_2_/CNT NNs composites exhibit high reversible capacity and stable cycle performance. Additionally, the plasma one-step synergy concept can effectively achieve a uniform load of SnO_2_ nanoparticles while constructing CNTs conductive network, which is possessed of environmentally friendly, time- and energy-saving advantages. Although SnO_2_-based materials are no longer new materials applied in LIBs, experimental results confirm that the DC arc-discharge plasma as a method has exhibited great potential for the synthesis of nanomaterials.

## 4. Conclusions

In this paper, we successfully synthesized the SnO_2_/CNT NNs composites for the first time via DC arc-discharge plasma; in this process, the construction of CNTs conductive network and the loading of SnO_2_ nanoparticles were realized simultaneously, this plasma one-step synergy concept is possessed of environmentally friendly, time- and energy-saving advantages compared with chemical synthesis. The SnO_2_/CNT NNs composites were applied in LIBs, showing high specific capacity and stable cycle performance. It can achieve a capacity of 472 mAh g^−1^ after 200 cycles at 100 mA g^−1^, which is due to the fact that the nanonest-like CNT conductive structure provides adequate room for the volume expansion and also helps to transfer the electrons. These results encourage further research in which the DC arc-discharge plasma method can be used for synthesizing energy storage materials.

## Data Availability

Data underlying the results presented in this paper are not publicly available at this time but may be obtained from the authors upon reasonable request.

## References

[1] Kang K., Meng Y.S., Bréger J., Grey C.P., Ceder G. (2006). Electrodes with high power and high capacity for rechargeable lithium batteries. Science.

[2] Zai J.T., Wang K.X., Su Y.Z., Qian X.F., Chen J.S. (2011). High stability and superior rate capability of three-dimensional hierarchical SnS_2_ microspheres as anode material in lithium ion batteries. J. Power Sources.

[3] Wu H.B., Chen J.S., Hng H.H., Lou X.W.D. (2012). Nanostructured metal oxide-based materials as advanced anodes for lithium-ion batteries. Nanoscale.

[4] Liu C.J., Huang H., Cao G.Z., Xue F.H., Camacho R.A.P., Dong X.L. (2014). Enhanced electrochemical stability of Sn-carbon nanotube nanocapsules as lithium-ion battery anode. Electrochim. Acta.

[5] Deng W.N., Chen X.H., Liu Z., Hu A.P., Tang Q.L., Li Z., Xiong Y.N. (2015). Three-dimensional structure-based tin disulfide/vertically aligned carbon nanotube arrays composites as high-performance anode materials for lithium ion batteries. J. Power Sources.

[6] Xu Y.H., Liu Q., Zhu Y.J., Liu Y.H., Langrock A., Zachariah M.R., Wang C.S. (2013). Uniform nano-Sn/C composite anodes for lithium ion batteries. Nano Lett..

[7] Zhen S., Yi H., Chen Y., Zhang X., Wang K., Chen R. (2015). Tin nanoparticle-loaded porous carbon nanofiber composite anodes for high current lithium-ion batteries. J. Power Sources.

[8] Zhang W.J. (2011). A review of the electrochemical performance of alloy anodes for lithium-ion batteries. J. Power Sources.

[9] Ashuri M., He Q.R., Shaw L. (2016). Silicon as a potential anode material for Li-ionbatteries: Where size, geometry and structure matter. Nanoscale.

[10] Shaw L., Ashuri M. (2019). Coating—A potent method to enhance electrochemical performance of Li(Ni_x_Mn_y_Co_z_)O_2_ cathodes for Li-ion batteries. Adv. Mater. Lett..

[11] Li Y., Yu S.L., Yuan T.Z., Yan M., Jiang Y.Z. (2015). Rational design of metal oxide nanocomposite anodes for advanced lithium ion batteries. J. Power Sources.

[12] Liang C., Gao M.X., Pan H.G., Liu Y.F., Yan M. (2013). Lithium alloys and metal oxides as high-capacity anode materials for lithium-ion batteries. J. Alloys Compd..

[13] Zhang C., Wang Z., Cui Y., Niu X.Y., Chen M., Liang P., Liu J.H., Liu R.J., Li J.C., He X. (2021). Dealloying-derived nanoporous Cu_6_Sn_5_ alloy as stable anode materials for lithium-ion batteries. Materials.

[14] Chu D.B., Li J., Yuan X.M., Li Z.L., Wei X., Wan Y. (2012). Tin-based alloy anode materials for lithium ion batteries. Prog. Chem..

[15] Jhan Y.R., Duh J.G., Tsai S.Y. (2021). Synthesis of confinement structure of Sn/C-C (MWCNTs) composite anode materials for lithium ion battery by carbothermal reduction. Diamond Relat. Mater..

[16] Luo Z.Y., Peng M.L., Lei W.X., Pan Y., Zou Y.L., Ma Z.S. (2019). Electroplating synthesis and electrochemical properties of CNTs/(Ni-P)/Sn as anodes for lithium-ion batteries. Mater. Lett..

[17] Huang L., Huang P., Chen P., Ding Y.L. (2020). Metal nanodots anchored on carbon nanotubes prepared by a facile solid-state redox strategy for superior lithium storage. Funct. Mater. Lett..

[18] Zhong Y., Li X.F., Zhang Y., Li R.Y., Cai M., Sun X.L. (2015). Nanostructued core–shell Sn nanowires @ CNTs with controllable thickness of CNT shells for lithium ion battery. Appl. Surf. Sci..

[19] Liu H.D., Huang J.M., Li X.L., Liu J., Zhang Y.X. (2012). SnO_2_ nanorods grown on graphite as a high-capacity anode material for lithium ion batteries. Ceram. Int..

[20] Wang F., Song X.P., Yao G., Zhao M.S., Liu R., Xu M.W., Sun Z.B. (2012). Carbon-coated mesoporous SnO_2_ nanospheres as anode material for lithium ion batteries. Scripta Mater..

[21] Kuriganova A.B., Vlaic C.A., Ivanov S., Leontyeva D.V., Bund A., Smirnova N.V. (2016). Electrochemical dispersion method for the synthesis of SnO_2_ as anode material for lithium ion batteries. J. Appl. Electrochem..

[22] Chen T.Q., Pan L.K., Liu X.J., Yu K., Sun Z. (2012). One-step synthesis of SnO_2_–reduced graphene oxide–carbon nanotube composites via microwave assistance for lithium ion batteries. RSC Adv..

[23] Sadakiyo M., Yoshimaru S., Kasai H., Kato K., Takata M., Yamauchi M. (2016). A new approach for the facile preparation of metal-organic framework composites directly contacting with metal nanoparticles through arc plasma deposition. Chem. Commun..

[24] Santhosh N., Filipič G., Tatarova E., Baranov O., Kondo H., Sekine M., Hori M., Ostrikov K., Cvelbar U. (2018). Oriented carbon nanostructures by plasma processing: Recent advances and future challenges. Micromachines.

[25] Tanaka M., Kageyama T., Sone H., Yoshida S., Okamoto D., Watanabe T. (2016). Synthesis of lithium metal oxide nanoparticles by induction thermal plasmas. Nanomaterials.

[26] Guo B., Košiček M., Fu J.C., Qu Y.Z., Lin G.H., Baranov O., Zavašnik J., Cheng Q.J., Ostrikov K., Cvelbar U. (2019). Single-crystalline metal oxide nanostructures synthesized by plasma-enhanced thermal oxidation. Nanomaterials.

[27] Wang C., Chen J.Z. (2015). Atmospheric-pressure-plasma-jet sintered nanoporous SnO_2_. Ceram. Int..

[28] Li S.L., He Y., Jing C.G., Gong X.B., Cui L.L., Cheng Z.Y., Zhang C.Q., Nan F. (2018). A novel preparation and formation mechanism of carbon nanotubes aerogel. Carbon Lett..

[29] Li S.L., Zhang C.Q., He Y., Feng M., Ma C., Cui Y. (2019). Multi-interpolation mixing effects under the action of micro-scale free arc. J. Mater. Process. Tech..

[30] Li S.L., Wang K., Feng M., Yang H.L., Liu X.Y., He Y., Zhang C.Q., Wang J.Y., Fu J.F. (2020). Preparation of light-transmissive conductive film by free arc dispersed carbon nanotubes and thermos compression bonding. Carbon Lett..

[31] Li S.L., Ci Y.D., Zhang D., Zhang C.Q., He Y. (2020). Free arc liquid-phase dispersion method for the preparation of carbon nanotube dispersion. Carbon Lett..

[32] Wang X., Fan H., Ren P., Li M. (2014). Homogeneous SnO_2_ core-shell microspheres: Microwave-assisted hydrothermal synthesis, morphology control and photocatalytic properties. Mater. Res. Bull..

[33] Mouyane M., Ruiz J.M., Artus M., Cassaignon S., Jolivet J.P., Caillon G., Jordy C., Driesen K., Scoyer J., Stievano L. (2011). Carbothermal synthesis of Sn-based composites as negative electrode for lithium-ion batteries. J. Power Sources.

[34] Marcinek M., Hardwick L.J., Richardson T.J., Song X., Kostecki R. (2007). Microwave plasma chemical vapor deposition of nano-structured Sn/C composite thin-film anodes for Li-ion batteries. J. Power Sources..

[35] Kim J.G., Nam S.H., Lee S.H., Choi S.M., Kim W.B. (2011). SnO_2_ nanorod-planted graphite: An effective nanostructure configuration for reversible lithium ion storage. Acs Appl. Mater. Int..

[36] Lian P.C., Zhu X.F., Liang S.Z., Li Z., Yang W.S., Wang H.H. (2011). High reversible capacity of SnO_2_/graphene nanocomposite as an anode material for lithium-ion batteries. Electrochim. Acta..

[37] Yao J., Shen X., Wang B., Liu H., Wang G. (2009). In situ chemical synthesis of SnO_2_-graphene nanocomposite as anode materials for lithium-ion batteries. Electrochem. Commun..

